# Cherenkov light emission in molecular radiation therapy of the thyroid and its application to dosimetry

**DOI:** 10.1364/BOE.448139

**Published:** 2022-03-23

**Authors:** Jigar Dubal, Pedro Arce, Christopher South, Lucia Florescu

**Affiliations:** 1 Centre for Vision, Speech and Signal Processing, University of Surrey, GU2 7XH, United Kingdom; 2 CIEMAT (Centro de Investigaciones Energéticas, Medioambientales y Tecnológicas), 28040 Madrid, Spain; 3 Department of Medical Physics, Royal Surrey County Hospital NHS Foundation Trust, Guildford GU2 7XX, United Kingdom; 4 J.Dubal@surrey.ac.uk; 5 l.m.florescu@surrey.ac.uk

## Abstract

Numerical experiments based on Monte Carlo simulations and clinical CT data are performed to investigate the spatial and spectral characteristics of Cherenkov light emission and the relationship between Cherenkov light intensity and deposited dose in molecular radiotherapy of hyperthyroidism and papillary thyroid carcinoma. It is found that Cherenkov light is emitted mostly in the treatment volume, the spatial distribution of Cherenkov light at the surface of the patient presents high-value regions at locations that depend on the symmetry and location of the treatment volume, and the surface light in the near-infrared spectral region originates from the treatment site. The effect of inter-patient variability in the tissue optical parameters and radioisotope uptake on the linear relationship between the dose absorbed by the treatment volume and Cherenkov light intensity at the surface of the patient is investigated, and measurements of surface light intensity for which this effect is minimal are identified. The use of Cherenkov light measurements at the patient surface for molecular radiation therapy dosimetry is also addressed.

## 1. Introduction

Cherenkov light emission in biological tissue during radiation therapy has attracted considerable interest recently. It has emerged as an important tool for applications, potentially enabling novel approaches to dosimetry and functional imaging [1,2]. Radiation therapy is a well-established treatment method for cancer and other diseases that uses high doses of radiation to destroy diseased cells. In this study, we address the emission of Cherenkov light in molecular radiation therapy (MRT). In MRT, the radiation dose is delivered via radio-pharmaceuticals administered orally or intravenously, that are designed to accumulate in the diseased tissue [3]. MRT is highly effective, if the prescribed radiation dose is delivered, cost-efficient, and in some instances, the only treatment option. In MRT, the radiation dose and Cherenkov light are produced by charged particles created as a result of the radioactive decay of the radio-pharmaceuticals. While all these charged particles deliver dose, Cherenkov light is generated only by the charged particles that have the phase velocity exceeding the phase velocity of light in the tissue [4]. In this study, we investigate the characteristics of Cherenkov light generated in MRT treatments of the thyroid, and explore the possibility of quantifying the dose absorbed by the tissue using measurements of Cherenkov light.

The effectiveness of radiation therapy depends on accurate assessment and control of the amount of radiation dose deposited at the treatment site. Due to the lack of control in the uptake, retention and distribution of the radio-pharmaceuticals within the tissue [5], in MRT it is not possible to accurately plan and control the delivered radiation dose prior to treatment, and the dose can only be assessed post radioisotope administration. In MRT, a prescribed amount of radio-pharmaceuticals (quantified by radioactivity, rather than radiation dose) is administered, and the same amount of radio-pharmaceuticals result in different dose at the treatment site for different patients, and therefore in different treatment outcomes. Thus, patient-specific techniques for dose characterisation (dosimetry) in MRT are very important for treatment assessment and adaptation and ultimately for increased treatment efficacy. Currently, only limited techniques are available for this purpose. Single Photon Emission Computed Tomography (SPECT) can be used to determine the distribution of the radio-pharmaceuticals, which can then be used for dose estimations [6]. However, such an approach can be costly and is therefore less available [7], and SPECT-based dosimetry still suffers from limited accuracy [8].

Cherenkov light emission in biological tissue containing radioisotopes or irradiated by external radiation beams, and the ability to measure the emitted light emerging from the tissue, have been demonstrated [1,2,9,10]. The intensity of Cherenkov light emission was demonstrated to be directly proportional to the absorbed dose in external-beam radiation therapy [1] and to the radioactivity in tissue containing radioisotopes [11]. Phantom and small-animal studies have demonstrated that Cherenkov light can be used to image the spatial distribution and concentration of therapeutic radionuclides [9,10,12]. Also, the use of Cherenkov light for real-time monitoring of the radiation dose delivered in external-beam radiation therapy has been demonstrated [13,14]. Nonetheless, Cherenkov light emission in MRT is still unexplored. Although Cherenkov light emission during MRT is reduced in comparison to external-beam radiation therapy, the ability to detect Cherenkov light emitted during MRT of the thyroid has been demonstrated experimentally [15]. However, these studies have focused only on the Cherenkov light reaching the surface of the patient, and the relationship between the measured light intensity and the dose absorbed by the tissue has not been addressed.

In this study, we perform numerical experiments using clinical patient Computed Tomography (CT) data to investigate Cherenkov light emission in MRT of hyperthyroidism [16] and papillary thyroid carcinoma (PTC) [17], the most common MRT treatments of the thyroid. In particular, we investigate in detail the spatial and spectral characteristics of Cherenkov light both inside the treated tissue and at the patient surface. The relationship between the intensity of Cherenkov light and the dose absorbed by the tissue is also investigated, as is the effect of inter-patient variability in tissue characteristics and radioisotope uptake on this relationship. Based on these results, we present a proposal for and preliminary assessment of a patient-specific dosimetry technique based on measurements of Cherenkov light at the patient surface, and show how the spatial and spectral characteristics of Cherenkov light can be exploited for an optimal implementation of this technique.

## 2. Method

We used Monte Carlo simulations and clinical data to characterise the emission and propagation of Cherenkov light within the body, as well as the dose deposition for MRT treatment of hyperthyroidism and PTC. Patient data was used to define the sample and consisted of CT images and corresponding patient structure (RTStruct) files in Digital Imaging and Communications in Medicine (DICOM) format. Additionally, the CT scanner calibration curve relating the image’s Hounsfield number (HU) with material density has been used. The CT images provided the 3-dimensional patient (sample) geometry and, together with the calibration curve, the material density. The RTStruct file provided segmentations of Volumes of Interest (VOI) (the treatment volumes, in this case) from the CT data, the thyroid gland and a region modeled as a tumour (within the thyroid volume), respectively.

Monte Carlo simulations were performed using the Geant4 toolkit - GAMOS (Geant4-based Architecture for Medicine-Oriented Simulations) [18], and the GAMOS tissue-optics package for light propagation in biological tissue [19]. The voxelised sample geometry was defined in GAMOS using the CT data. As in clinical situations [20], the radioisotope Iodine- 131 ( I131 ) was utilised for simulating delivery of the radiation dose. This radioisotope undergoes beta decay, producing beta particles (electrons) responsible for dose deposition and Cherenkov light generation in regions local to the concentration of radioisotope. In addition, some gamma radiation is also produced; this can, in turn, generate charged particles that could produce Cherenkov light and deposit radiation dose.

### 2.1 Simulation sample

A geometry file was created based on the CT data that provides the HUs within the geometry. The geometry voxel size was provided by the CT data and was 0.98mm x 0.98mm x 1.25mm . For use in GAMOS, the HU of each voxel was converted to material type. Specifically, by using the scanner calibration curve, the HU values of each voxel in the 3D CT image were converted to material density ( $\text{g}/{\text{cm}}^{3}$ ), which was used to define the material type. The following materials were used: adipose tissue, muscle, bone, air, and soft tissue. The soft tissue was considered to accurately represent the thyroid gland. MRT treatment of hyperthyroidism and PTC was simulated by distributing the radioisotope in the respective treatment volumes, i.e., the entire thyroid gland for hyperthyroidism and only the tumour for PTC. Additionally, a second scenario for the PTC treatment was considered, with some radioisotope accumulation also in the surrounding thyroid tissue, in addition to the tumour. The treatment volumes for both treatments are presented in some of the figures in Section 3. For each study, a different set of clinical CT data was used. Along a line from the centre of the VOIs to the surface of the sample, the thicknesses of the skin, adipose, muscle, and thyroid tissue are approximately 2 mm, 4 mm, 10 mm, and 8 mm respectively for the patient used for the study of hyperthyroidism, and 2 mm, 10 mm, 4 mm and 4 mm, respectively for the patient used for the study of PTC. The clinical CT data used in the study for PTC did not include a tumour. To represent a tumour, a spheroid with a maximum diameter of 1.2 cm and a volume of 0.85 ${\text{cm}}^{3}$ positioned at (x= 15.6 mm, y= −43.9 mm, z= −75.6 mm) was contoured on the image, and the CT number within this segment was altered to display a contrast relative to that of the thyroid. Specifically, the HU within this structure was set to 41% of that of the thyroid gland to indicate the existence of a tumour [21]. This structure was also assigned different optical properties, as described below.

In order to simulate emission and propagation of Cherenkov light, each tissue type (material) in the patient geometry was characterised by spectrally-dependent optical absorption and scattering coefficients, anisotropy factor, and refractive index; these were incorporated into the geometry within GAMOS. The spectral range used in this study was 500−1200 nm, covering the broad emission spectrum of Cherenkov light and including both the visible light region as well as the near-infrared (NIR) region, where light absorption is minimal in biological tissue [2]. Although Cherenkov light is predominantly emitted in the ultraviolet region, the absorption of biological tissue at these wavelengths is large and for this reason, the spectral range beyond the ultraviolet region has been considered.

The absorption and scattering coefficients of thyroid tissue were obtained from literature [22]. The absorption coefficients of skin, adipose, muscle, and bone were calculated based on the relative contribution of chromophores within the tissue as [23]

(1) $$\mu_{\text{a}}(\lambda )=\left[{\text{SO}}_{2}\mu_{{\text{HbO}}_{2}}(\lambda )+\left(1-{\text{SO}}_{2}\right)\mu_{\text{Hb}}(\lambda )\right]+\text{W}\mu_{{\text{a, H}}_{2}0}(\lambda )+\text{F}\mu_{\text{a, fat}}(\lambda )+\text{M}\mu_{\text{a, mel}}(\lambda ).$$

Here λ is the wavelength, ${\text{SO}}_{2}$ is the oxygen saturation, and $\mu_{\text{Hb}O_{2}/\text{Hb}}$ are the absorption coefficients of oxygenated and deoxygenated hemoglobin, respectively. W, F, and M, and $\mu_{{\text{a,H}}_{2}0}$ , $\mu_{\text{a,fat}}$ , $\mu_{\text{a,mel}}$ are the fractional components and absorption coefficients of water, fat, and melanin, respectively. All these parameters were obtained from literature [24–32].

The scattering coefficient $\mu_{s}$ of each material was obtained using the reduced scattering coefficient $\mu_{\text{s}}^{\prime }$ and anisotropy factor g as [23]

(2) $$\mu_{\text{s}}=\mu_{s}^{\prime }{\left(1-g\right)}^{-1}.$$

The reduced scattering coefficient of thyroid tissue was obtained from literature [22], while that of the skin, adipose, muscle, and bone tissues was calculated by considering the contributions from Mie and Rayleigh scattering as [24]

(3) $$\mu_{s}^{\prime }(\lambda )=a\left[f_{\text{Ray}}{\left(\frac{\lambda }{\lambda_{0}}\right)}^{-4}+(1-f_{\text{Ray}}){\left(\frac{\lambda }{\lambda_{0}}\right)}^{-b_{\text{Mie}}}\right].$$

**Fig. 1.**
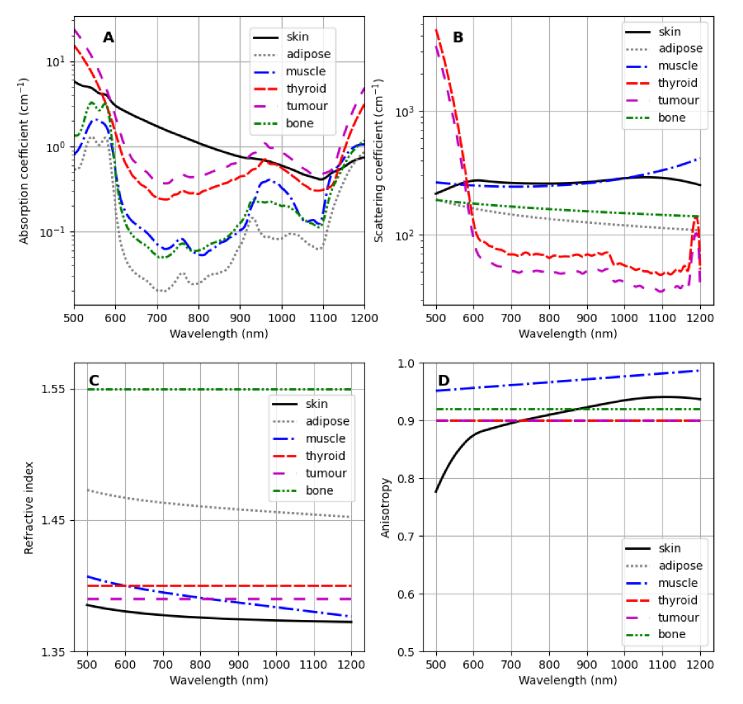
Optical properties of biological tissue used in this study: A) absorption coefficient $\mu_{a}$ ; B) scattering coefficient $\mu_{s}$ ; C) refractive index n ; and D) anisotropy factor g , where the graphs for thyroid, tumour and adipose tissue overlap at g=0.9 .

Here, a is the scattering amplitude and represents the reduced scattering coefficient at $\lambda_{0}$ , $f_{\text{Ray}}$ is the fractional component of Rayleigh scatter, $b_{\text{Mie}}$ is the scatter power, and λ is the wavelength (nm). The quantities a , $f_{\text{Ray}}$ and $b_{\text{Mie}}$ for each of the tissue constituents used in this study have been obtained from tabulated data [24]. The anisotropy factor g of bone, skin, and muscle tissue was also obtained from literature [24,33–35], while the average scattering anisotropy of biological tissue ( g=0.9 ) was used for the thyroid and adipose tissue [36]. The refractive index of adipose tissue and skin was determined by applying the Sellmeier formula [37] and Cauchy dispersion equation [38] respectively, using experimentally-determined coefficients [39]. The refractive index of muscle tissue was obtained from previous work [40], while the refractive index of bone and thyroid tissue used in the simulations was 1.55 and 1.40 , respectively, as indicated by experimental data [41,42]. The tumour volume was considered to be characterised by optical absorption and scattering coefficients that differ from those of the healthy tissue by +44% and −30% , respectively [43], a refractive index of n=1.39 [44], and the average anisotropy in biological tissue ( g=0.9 ). We have neglected dispersion as it is more pronounced at shorter wavelengths [45], a spectral region in which the emitted light is strongly absorbed by the biological tissue such that it has minimal contribution to the surface signal. In the spectral region of interest of 600−900 nm, the largest variation in the refractive index value relative to the value at 750 nm is of about 0.6% [45], giving rise to variations of less than 3% in the emitted Cherenkov signal. Figure 1 presents the optical properties of the relevant tissue types used in this study.

The inter-patient variability of tissue optical parameters was obtained from literature [43,44,46– 49]. Table 1 presents the largest percentage variability of the optical parameters relative to the values presented in Fig. 1, which are considered as baseline values. We have neglected the variability in the optical parameters of skin, given that skin is about 2 mm thick and patient variability has minimal impact on light transport. Similarly, we have neglected the variability in bone, which is not present near the thyroid gland or along the path of light propagation towards the patient surface. To represent the greatest impact of inter-patient variability on light transport in tissue, we considered simultaneous increase or simultaneous decrease of all the parameters relative to baseline values.

**Table 1.** Inter-patient optical parameter variability for the types of biological tissue considered in this study, showing the percentage variation relative to the values presented in Fig. 1.

	Percentage variation (%)				
Tissue	$\mu_{\text{a}}$	$\mu_{\text{s}}$	n	g	Ref.
Adipose	±9.9	±8.9	±0.6	0	[46], [47]
Muscle	±9.9	±8.9	±1.1	0	[46], [47]
Thyroid	±5.8	±3.0	±2.0	±2.15	[49], [44], [48]
Tumour	±6.6	±4.6	±2.0	0	[43], [44]

The radioisotope was distributed within the treatment volume only. The values used for the radioisotope I131 activity correspond to clinical values, which are in the range of 100 MBq - 700 MBq for treatment of hyperthyroidism [50], and in the range of 300 MBq - 1.1 GBq for the treatment of PTC [51]. In the two PTC cases where accumulation of the radioisotope in the thyroid is also considered, 90% and 75% of the total radioisotope uptake is in the tumour, and 10% and 25% , respectively, in the thyroid gland.

### 2.2 Monte Carlo simulations

In this study, the following processes have been simulated using GAMOS: the radioactive beta decay of the radioisotope I131 ; the production and propagation of radioactive decay products (including beta particles and gamma radiation); dose deposition; Cherenkov light emission and transport within the biological tissue; and Cherenkov light emerging from the patient surface. Each simulated event in GAMOS represents a single isotope disintegration. 2 x $10^{10}$ events were simulated and the data output was averaged per simulated event.

The number of Cherenkov photons generated per unit length and per unit wavelength was calculated within GAMOS using the Frank-Tamm formula [52]

(4) $$\frac{d^{2}N}{dxd\lambda }=\frac{2\pi z^{2}}{137}\left(1-\frac{1}{\beta {\left(E\right)}^{2}n^{2}}\right)\frac{1}{\lambda^{2}}$$

where λ is the photon wavelength, where z and n are respectively the atomic number and refractive index of the medium, and β is the ratio between the phase speed of the charged particle and the speed of light in a vacuum. The probability of absorption and scattering of Cherenkov light was stochastically sampled within GAMOS to simulate light-tissue interactions. By tracking the Cherenkov light propagation through the geometry, the light intensity at the surface of the sample was determined, together with the positions within the sample where the photons contributing to this are emitted.

Geant4’s ability to model charged particle transport enables quantification of the dose deposited within the voxelised geometry. Specifically, through integrated modelling of radiation transport, Coulomb interactions and other collisional energy losses were considered. In the process leading to dose deposition, charged particles, as they propagate, transfer their kinetic energy to the medium and lose some energy through radiative losses such as brehmsstrahlung radiation. The energy deposited by charged particles to a voxel was summed to determine the total energy absorbed by that voxel. The voxelised absorbed dose in units of Gray (Gy) was determined by dividing the deposited energy by the voxel mass, which is determined from the voxel volume and density. All dose calculations were performed by GAMOS.

**Fig. 2.**
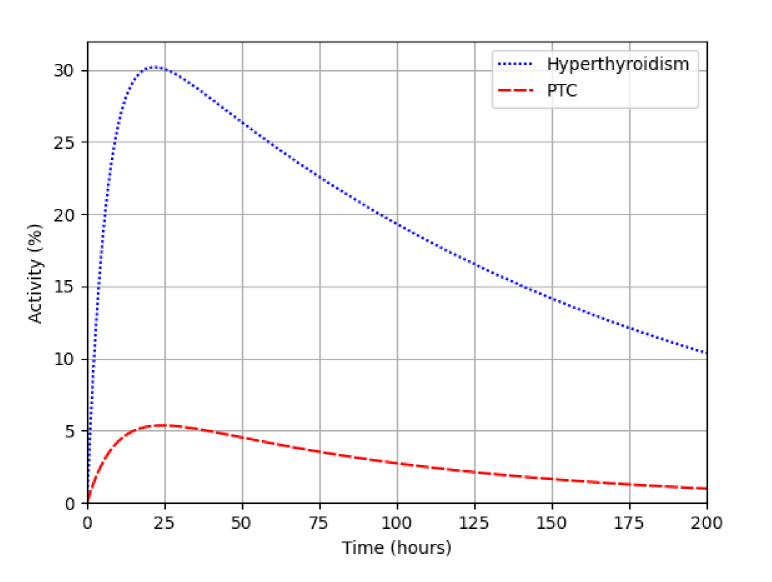
The activity (as a percentage of the administered activity) as a function of time for the treatment of hyperthyroidism (dotted) and PTC (dashed).

For a given radioisotope activity, the result obtained from Monte Carlo simulation (averaged per disintegration) was multiplied by the total number of disintegrations corresponding to the decay of the administered activity. This was in turn determined by integrating over time the time-dependent activity. In this study, the radiopharmaceutical biokinetics were represented by a biphasic model taking into account both the uptake and elimination of the radiopharmaceutical [53]. Using this model, the activity A as a function of time t follows the equation

(5) $$A(t)=\eta A_{0}(2^{-t/T_{\text{eff}}}-2^{-t/T_{\text{a}}}),$$

where $A_{0}$ is the administered activity, η describes the average intake by the treatment volume, and the parameters $T_{\text{eff}}$ and $T_{\text{a}}$ correspond to the loss and influx of the radioisotope into the treatment volume, respectively. In this study, we have considered η=30% , $T_{\text{eff}}=111.4$ hours, and $T_{\text{a}}=4.5$ hours for the treatment of hyperthyroidism, and η=5.4% [54], $T_{\text{eff}}=68$ hours, and $T_{\text{a}}=6.5$ hours for the treatment of PTC. The values of $T_{\text{eff}}$ and $T_{\text{a}}$ have been obtained by fitting the biphasic model to existing uptake measurements for each type of treatment [55,56]. The time-activity curves for both treatments are displayed in Fig. 2.

For Cherenkov light intensity measurements, we have considered a measurement time of 20 minutes immediately after the time of maximum activity. Correspondingly, the number of disintegrations responsible for Cherenkov light emission was calculated by integrating A(t) given by Eq. (5) between t=24 hours and t=24h+20 minutes, for hyperthyroidism, and between t=25 hours and t=25h+20 minutes for PTC. In both cases, the number of disintegrations responsible for dose delivery were calculated by setting the integral limits to t=0 and t=∞ .

## 3. Results

### 3.1 Cherenkov light intensity and dose within the patient

**Fig. 3.**
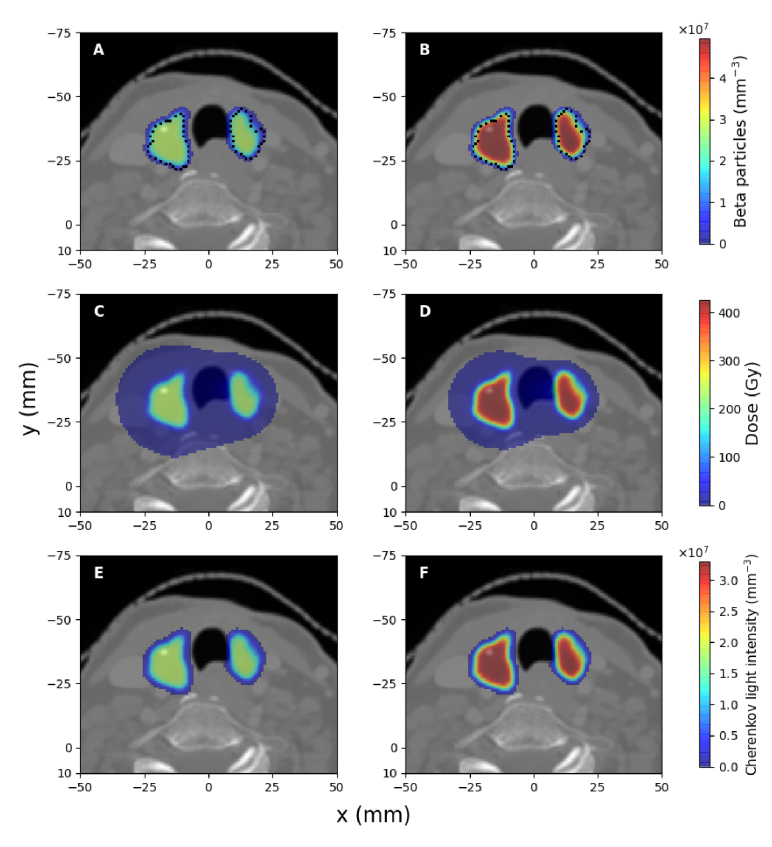
The spatial distribution, of all beta particles produced from radioactive decay of I131 (A,B), absorbed dose (C,D), and spectrally-integrated emitted Cherenkov light intensity (E,F), for the treatment of hyperthyroidism, for administered activities of 400 MBq (left column) and 700 MBq (right column). A dotted contour of the treatment volume is also presented in A and B. All results are displayed in the trans-axial plane z=−50.6 mm of the CT data used.

**Fig. 4.**
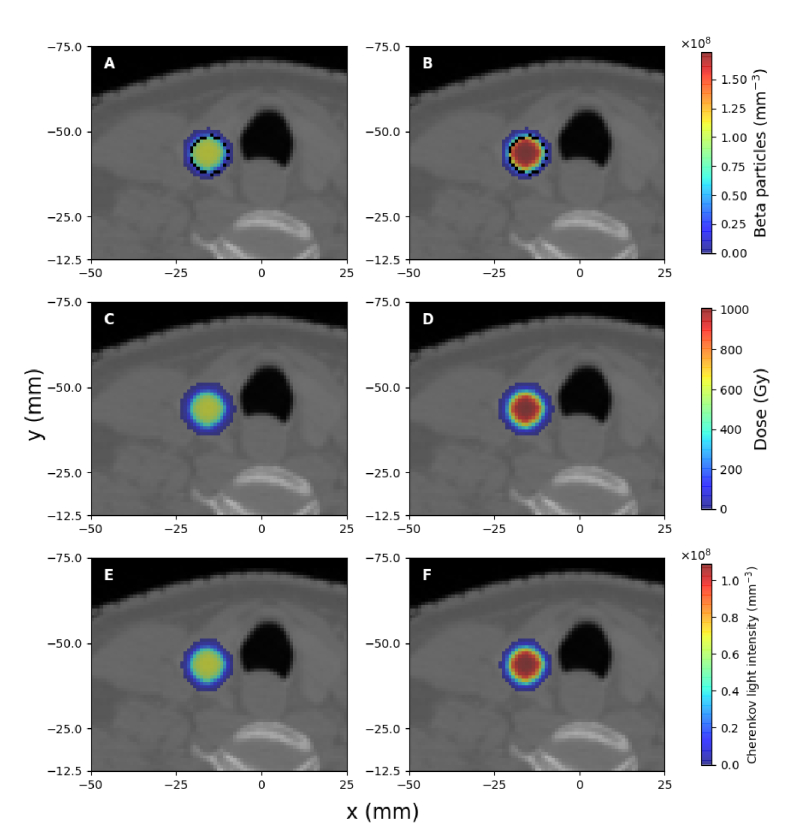
The same as for Fig. 3 but for the treatment of PTC with all the radioisotope uptake distributed within the tumour, and administered activities of 700 MBq (A,C,E) and 1.1 GBq (B,D,F), in the trans-axial plane z=−74.4 mm. A dotted contour of the tumour is presented in A and B.

**Fig. 5.**
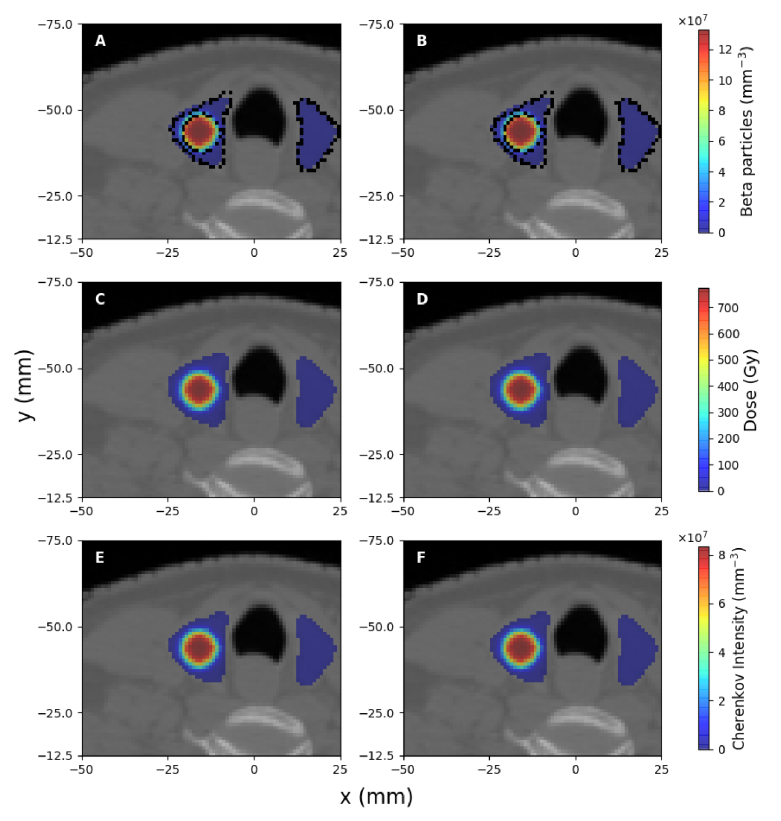
The same as for Fig. 4 but for 75% of the radioisotope uptake retained by the tumour and 25% by the thyroid. Dotted contours of the tumour and the thyroid are also presented in A and B.

The spatial distribution of beta particles produced as a result of radioactive decay, the deposited dose, and the spectrally-integrated intensity of emitted Cherenkov light in the trans-axial plane containing the centre of the treatment volume are presented in Fig. 3, 4 and 5 for the treatment of hyperthyroidism and PTC, respectively, for clinically-relevant values of radioisotope activities and different tumour uptakes, in the case of PTC. Due to the random propagation directions of beta particles produced by radioactive decay, Cherenkov light emission is isotropic. The displayed Cherenkov light intensity corresponds to the emission in all directions (the total number of photons emitted in all directions). In these images, in order to remove statistical noise, threshold filtering was applied to remove signals less than 1% of the average signal within the treatment volumes. The distribution of Cherenkov light and dose outside the treatment volume (where beta particles are produced) is consistent with the range in biological tissue of beta particles produced from I131 decay, which is about 2.5 mm [57]. The somewhat more spread dose distribution, particularly for hyperthyroidism, can be explained by the fact that dose deposition is also possible due to brehmsstrahlung radiation produced by beta particles interacting with the biological tissue, and because the decay of I131 also results in gamma radiation (predominantly at the energy of 0.364 MeV, with the energy 0.723 MeV also present [58]). These gamma photons can interact with biological tissue to produce secondary electrons that are responsible for dose deposition [59] in regions beyond the treatment volume, but most of these do not produce Cherenkov light. The linear attenuation coefficient of soft tissue at 0.364 MeV and 0.723 MeV is about 0.1 ${\text{cm}}^{-1}$ and, as such, secondary electrons can be produced and deposit dose at distances in the order of centimetres from the location where gamma radiation is produced. However, the majority of these secondary electrons have an energy (transferred via Compton scattering) below the threshold of 0.211 MeV for Cherenkov light production in biological tissue. We note that we did observe Cherenkov photons emitted outside the treatment volume, in particular close to the surface, but the corresponding intensity is below 1% of the average intensity and is not displayed in these figures. We also note that for the hyperthyroidism treatment, central to the two thyroid lobes (in the trachea region), while there is deposited dose, the Cherenkov light intensity is negligible. This is because this region was modelled as air, and the small refractive index results in a very low emission intensity. However, consistent with the relatively lower probability of gamma emission, the dose absorbed by tissue beyond the treatment volume is significantly low, with only sizable values occurring between the thyroid lobes where the contributions from radioactivity in the two lobes overlap. For the treatment of PTC with all of the radioisotope uptake distributed in the tumour, the dose deposited more than a few millimetres beyond the tumour is less than 1% of the maximum absorbed dose in the tumour; this was removed through threshold filtering and is not displayed in Fig. 4. For the PTC cases where there is radioisotope uptake also by the thyroid gland, the average absorbed dose by the thyroid gland is about 3% of the dose absorbed by the tumour, while the absorbed dose beyond the thyroid gland is below the 1% threshold and is not displayed in Fig. 5.

**Fig. 6.**
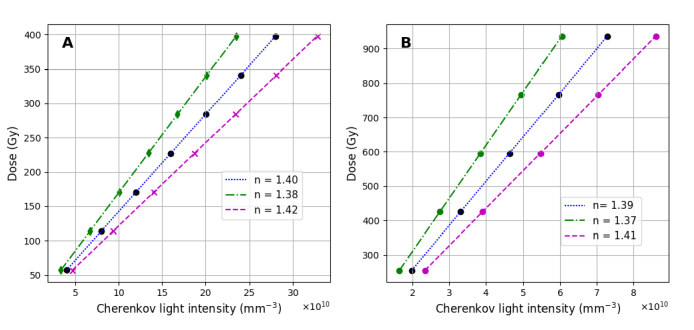
The mean deposited dose in the thyroid and tumour in the treatment of hyperthyroidism (A) and PTC (with 100% of the radioisotope uptake distributed in the tumour) (B), respectively, as a function of the total Cherenkov light intensity in the treatment volume. The three sets of data correspond to three different values of the refraction index n of the thyroid and tumour, respectively.

Figure 6 presents the radiation dose as a function of the emitted Cherenkov light intensity, showing, as expected, a linear relationship since both dose and Cherenkov light intensity depend linearly on the radioactivity. The emitted Cherenkov light intensity is also determined by the tissue refractive index, as expressed by Eq. (4); this can vary between patients but it does not affect dose deposition. This results in different linear relationships between the Cherenkov light intensity and the deposited dose for different patients. We obtain that for the range of clinically relevant radioisotope activities and refractive index values, for the same administered activity and deposited dose, a variation of about 2% in the refractive index of thyroid or tumour tissue can lead to a variation in Cherenkov light intensity of up to 35% .

### 3.2 Cherenkov light intensity at the surface

**Fig. 7.**
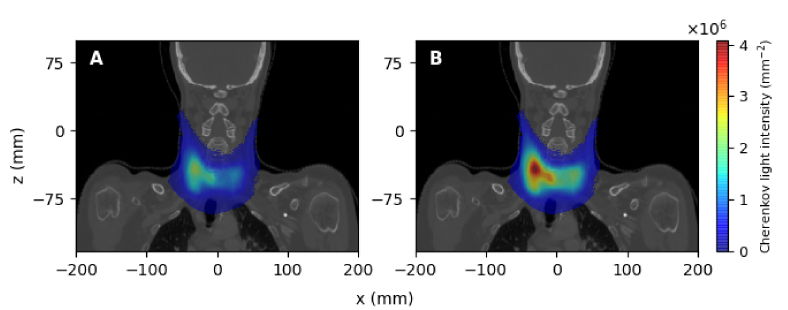
Spectrally-integrated Cherenkov light intensity emerging from the patient surface for the treatment of hyperthyroidism, for administered activities of A) 400 MBq and B) 700 MBq.

**Fig. 8.**
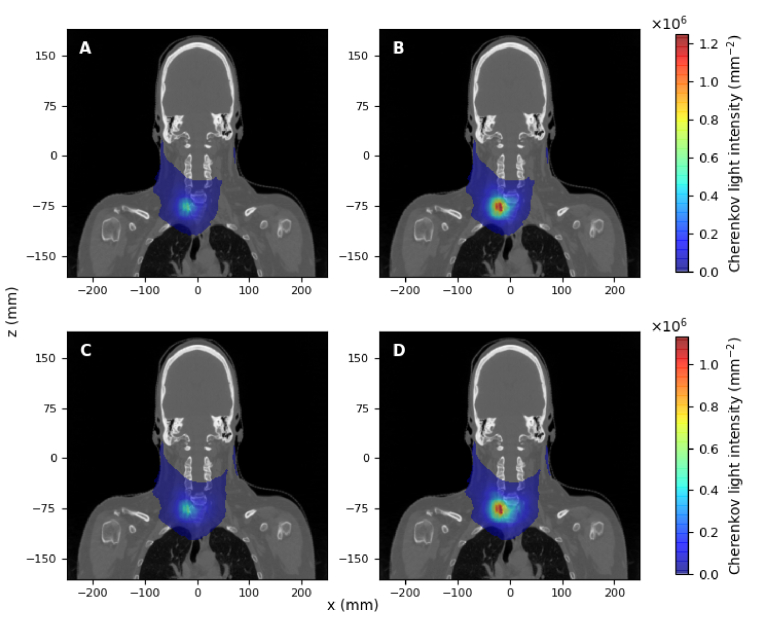
Spectrally-integrated Cherenkov light intensity emerging from the patient surface for the treatment of PTC, for administered activities of (A,C) 500 MBq and (B,D) 1.1 GBq. (A,B) correspond to 100% of the radioisotope uptake distributed in the tumour, and (C,D) to 75% of the radioisotope uptake distributed in the tumour and 25% in the thyroid.

The spatial distribution of the spectrally-integrated Cherenkov light intensity on the surface is displayed in Fig. 7 and 8 for the two treatments. In both cases, the light intensity displayed corresponds to the photons propagating in all directions. In these images, the heat-map is overlaid on a CT slice best representing the neck area ( y=−16.1 mm) in the coronal plane for anatomical reference. For the treatment of hyperthyroidism, while relatively high values of Cherenkov light intensity are obtained central to the two thyroid lobes, light intensity on the patient surface has the highest value on one side of the patient. This is due to a larger lobe volume containing radioisotopes on that side for the particular patient used in our study, resulting in higher Cherenkov light emission on that side. For the treatment of PTC, the region of high Cherenkov light intensity on the patient surface is localised directly above the thyroid lobe that encapsulates the tumour. In the PTC case with radiosiotope uptake also by the thyroid gland, relative to the case of the radioisotope uptake entirely by the tumour, the hot-spot intensity is slightly decreased (by about 7 %) and light intensity distribution is slightly extended, but by values of under 6% of the maximum value.

**Fig. 9.**
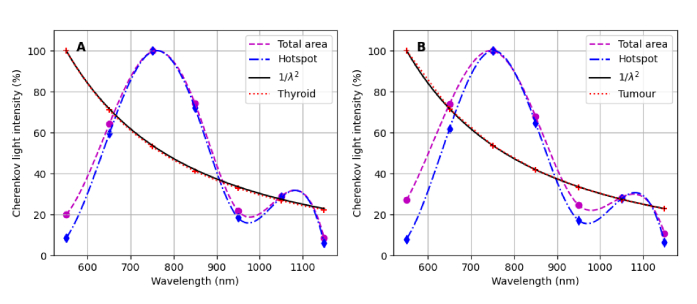
The normalized spectrum of Cherenkov light within the treatment volume and for light emerging from the hot spot and the entire surface, for (A) treatment of hyperthyroidism for an administered activity of 500 MBq, and (B) for PTC treatment for an administered activity of 700 MBq. In B, the spectra corresponding to different tumour uptakes overlap.

Based on the results presented in Fig. 7 and 8, we have considered surface measurements of total Cherenkov light intensity emerging from the entire surface, as well as measurements restricted to the hot-spot regions. For the measurements on the entire surface, Cherenkov light intensity was spatially integrated over the entire area where photons were detected, while for measurements of light emerging from the hot-spot regions, a 25mm x 25mm window (centred at the hot-spot) was used for both cases. Figure 9 presents the spectrum of spatially-integrated Cherenkov light within the treatment volume at the hot spots and on the entire surface. We find that the emitted Cherenkov light is dominant in the visible region and is in accord with the Frank-Tamm formula (Eq. (4)). On the other hand, the spectral characteristics of light at the surface are determined by the spectral characteristics of the emitted light, by the thickness and spectral characteristics of the optical properties of various tissue components through which light propagates before reaching the surface, and by their relative values. Consistent with the tissue optical absorption spectrum, light emerging from the surface exhibits two spectral maxima and is dominant in the NIR region, where tissue absorption is minimal.

**Fig. 10.**
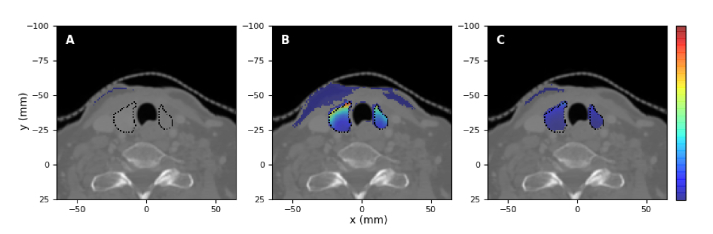
Hyperthyroidism treatment. The normalised spatial distribution of the emitted Cherenkov photons (for all activities) contributing to the total surface intensity, for the wavelength intervals (A) 500−600 nm, (B) 600−900 nm and (C) 900−1200 nm. The figures are in the trans-axial plane z=−50.6 mm, and the number of photons in each figure was normalised to the maximum value in the 600−900 nm range. A dotted contour of the treatment volume is also presented.

**Fig. 11.**
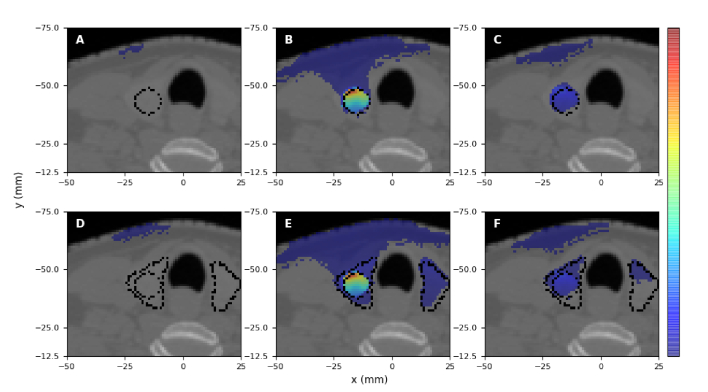
PTC treatment. The normalised spatial distribution of the emitted Cherenkov photons (for all activities) contributing to the total surface intensity, for the wavelength intervals (A,D) 500−600 nm, (B,E) 600−900 nm, and (C,F) 900−1200 nm. A,B,C correspond to the entire radioisotope uptake distributed in the tumour, and (D,E,F) to 75% of the radioisotope uptake in the tumour and 25% in the thyroid. The figures are in the trans-axial plane z=−74.4 mm, and the values in each figure have been normalised to the maximum value in the 600−900 nm range for each treatment. Dotted contours for tumour (A-C) and for tumour and thyroid (D-E) are also shown.

Figures 10 and 11 display the normalised distribution of emitted Cherenkov photons that contribute to the total (spatially-integrated over the whole surface) light intensity emerging from the patient surface, for various spectral intervals. This result is the same for all administered activities. We find that in the 600−1200 nm spectral interval, the surface light mostly originates in the treatment volume, which is where the radioisotope source is located and where most Cherenkov light is produced. There is also some very small contribution from light generated close to the surface (by secondary electrons generated by the gamma photons that result from the radioisotope decay). This contribution is larger in the case of PTC treatment due to the greater thickness of the adipose tissue (which has a larger refractive index), resulting in more Cherenkov photons being emitted close to the surface. Due to the strong absorption of tissue in the spectral region of 500−600 nm, photons at these wavelengths emitted in the treatment volume do not reach the surface, and the light generated close to the surface entirely determines the surface intensity in this spectral region. We note that in contrast to Fig. 3 and 4, Cherenkov light emitted close to the surface is visible in Fig. 10 and 11 since although emission is much higher in the treatment volume, only a small fraction of this emitted light reaches the surface, resulting in the increased relative value in Fig. 10 and 11 of the intensity of Cherenkov light emitted closer to the surface.

**Fig. 12.**
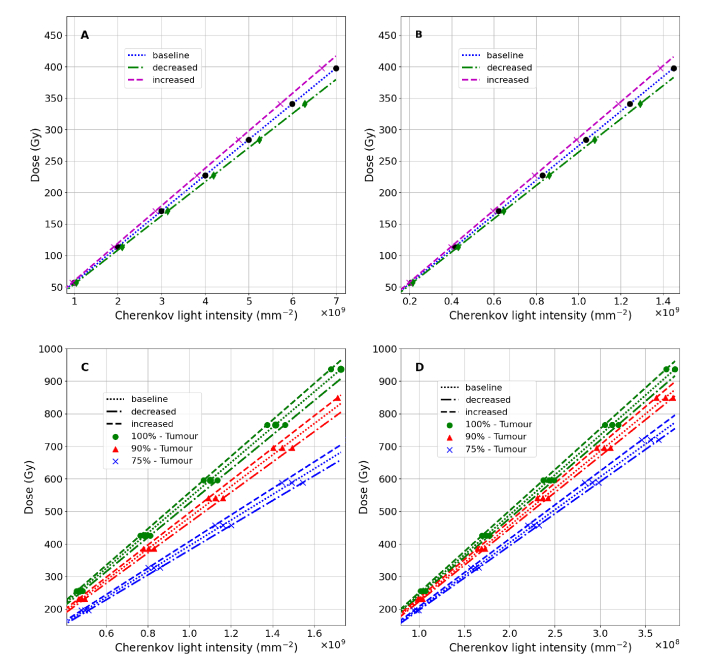
The mean dose deposited in the treatment volume as a function of the spatially and spectrally-integrated Cherenkov light surface intensity, for measurements on the entire surface (A, C) and at the hot spot (B,D), for the treatment of hyperthyroidism (A,B) and PTC (C,D) where 100% , 90% and 75% of the radioisotope uptake is distributed within the tumour and the rest in the thyroid. The three sets of data for each case correspond to the baseline set of optical parameters and to optical parameters exhibiting an increase and decrease with respect to the baseline values (as presented in Table 1).

The relationship between the deposited dose in the treatment volume and the total (spectrally- and spatially-integrated) light intensity for each type of light intensity measurement is presented in Fig. 12 for the baseline set of optical parameters, as well as for optical parameters presenting the largest deviations (increase and decrease) from the baseline values. We observe that the variability in the linear relationships between the deposited dose and the measured Cherenkov light intensity, which results from the variability in the optical parameters of the tissue, is reduced in the case of detection at the hot spot region, compared with measurements over the whole area, and for PTC treatment compared to hyperthyroidism treatment. This variability is determined by the thickness of different tissue types and their effective optical attenuation coefficients and its variability. Light detected on the entire surface propagates through more tissue overall, and in the case of hyperthyroidism, through more muscle tissue (characterised by larger attenuation and greater inter-patient variability) than the case of PTC, and is therefore more affected by variations in tissue optical properties. Moreover, in the case of PTC, different linear relationships are obtained for different radioisotope uptakes by the tumor and thyroid tissue, and this variability is reduced for light measurements at the hot spot.

## 4. Discussion and conclusion

Numerical experiments were performed to investigate the characteristics of Cherenkov light and dose deposition in MRT treatment of the thyroid, and the possibility of exploiting these characteristics for MRT dosimetry.

This study confirms that the emitted Cherenkov light is localised in the treatment volume and that its distribution mostly overlaps with that of the deposited dose. Its spectrum is consistent with the Frank-Tamm formula, and its intensity increases with the refractive index of the biological tissue and varies linearly with the deposited dose. On the other hand, the surface light is dominant in the NIR spectral region, and its spatial characteristics depend on the position and symmetry of the treatment volume. Light at the surface in the NIR spectral region originates mainly from the treatment volume, while for the spectral region below 600 nm, the surface light originates outside the treatment volume and is close to the surface. For the patient geometries considered in this study, light reaching the surface is localised on the side of the patient containing more of the treatment volume or its entirety. Furthermore, the linear relationship between the absorbed dose and the light intensity measured at the hot spot is more robust to inter-patient variability of tissue optical characteristics than for whole surface measurements.

The linearity between Cherenkov light intensity at the patient surface and the administered activity and deposited dose could pave the way to patient-specific dosimetry of MRT based on Cherenkov light measurements. Inter-patient variability of tissue optical parameters, variability in radioisotope uptake by the treatment volume, and the ability to detect light that originates from the treatment volume (the region of interest for dose deposition) are among the factors that affect the accuracy of this dosimetry method. An in-depth analysis of the accuracy of MRT dosimetry based on Cherenkov light measurements is beyond the scope of this study. Here we have considered the effect of inter-patient optical parameter variability and measurement type, as well as a preliminary study of the effect of variability in radioisotope uptake by the tumour, for PTC treatments. The first two columns in Table 2 present the maximum errors in dose estimations if a dose-light intensity calibration curve corresponding to the baseline set of optical parameters is used, for the treatments of hyperthyroidism and PTC with all the activity distributed in the tumour, respectively. Similar (but sightly larger) values were obtained for this type of error for PTC treatments with uptake by the thyroid too. The last two columns in Table 2 present the errors in tumour dose estimation for PTC treatments where radioisotopes are also distributed in the thyroid and for baseline optical parameters, if a calibration curve corresponding to 100% uptake by the tumour and baseline optical parameters is used. While these errors remain large, the technique could potentially still be used for gross-error detection in some cases.

**Table 2.** Uncertainty for absorbed dose estimates based on Cherenkov light measurements at the patient surface due to (i) inter-patient variability in tissue optical parameters, for the treatment of hyperthyroidism and PTC (with 100% of the uptake distributed in the tumour) with respect to the baseline set of optical parameters, and (ii) uptake variability, with respect to 100% of the uptake distributed in the tumour. The positive and negative values when considering the inter-patient variability in tissue optical parameters correspond to the largest and smallest optical tissue parameters, respectively.

	Dose uncertainty (%)			
	Due to tissue optical parameter variability		Due to uptake variability	
	
Area, Spectral range	Hyperthyroidism	PTC	PTC	PTC
		(100%)	(90%)	(75%)
Total Surface, 500-1200 nm	+4.8, -4.6	+2.9, -3.1	11	27
Total Surface, 700-800 nm	+4.7, -4.2	+2.7, -2.7	12	30
Hot spot, 500-1200 nm	+4.5, -3.7	+2.6, -2.2	6.7	17
Hot spot, 700-800 nm	+3.1,-2.1	+1.1,-0.3	6.5	17

Based on the results presented in Table 2, to achieve the best accuracy in dose estimation, surface light measurements are recommended over an optimal area at the hot spot (to reduce the effect of inter-patient variability of optical parameters) and in the NIR spectral range (to minimise the detection of light emitted outside of the treatment volume). Experimental studies [15] have demonstrated that measurements of surface light are possible. Surface light in the central region in Fig. 7 and 8 is likely to be observed by a camera positioned in the direction of the reader, but some of the light on the side may be missed. The position of the hot spot could be determined through preliminary scanning of the whole surface with cameras at different positions.

Based on the model (Eq. (5)) used for radiopharmaceutical biokinetics, the total number of disintegrations (producing the total dose) is directly proportional to $\eta (T_{\text{eff}}-T_{\text{a}})≃\eta T_{\text{eff}}$ , for $T_{\text{a}}\ll T_{\text{eff}}$ . On the other hand, for a light measurement time window $\Delta t_{m}\ll T_{\text{a}},T_{\text{eff}}$ , the number of disintegrations producing Cherenkov light is directly proportional to $\eta (T_{\text{eff}}2^{-t_{1}/T_{\text{eff}}}-T_{\text{a}}2^{-t_{1}/T_{\text{a}}})≃\eta T_{\text{eff}}$ , for $T_{\text{a}}\ll t_{1}\ll T_{\text{eff}}$ , where $t_{1}$ is the time after radioisotope administration when light measurements begin. Therefore, while the radiopharmaceutical biokinetics does determine the amount of deposited dose and the Cherenkov light intensity, its inter-patient variability does not strongly affect the linear relationship between these quantities and therefore does not strongly affect the dosimetry accuracy. Regarding the values of the dose and light intensity, in this study, the biphasic model (Eq. (5)) was fitted to one set of experimental measurements to determine the model parameters. As such, the results presented here for absorbed dose and light intensity are within the error associated with the patient variability of these parameters (which is roughly the sum of the errors in η and $T_{\text{eff}}$ ). We also note that the radioactive decay of I131 does not depend on the environment [60] and, as such, the decay products (leading to dose deposition and Cherenkov light emission) are the same for all patients for the same radioactivity intake. The variability in the tissue density and therefore the stopping power [61] could lead to variability in dose deposition, but this has the same effect on the accuracy of any dosimetry technique. Although possibly not having a strong effect for thyroid treatments, another factor leading to errors in dose estimation could be the patient geometry variability [62]. This can be addressed by performing Monte Carlo simulations with multiple patient CT data, as well as by experimental calibration measurements with multiple phantom geometries to obtain a multi-patient average of the dose-light intensity curve that accounts for this variability.

While additional studies are needed to establish and optimise the performance of a dosimetry technique based on Cherenkov light measurements, we note that current MRT dosimetry methods (involving SPECT) are characterised by an uncertainty in dose estimates of 14−102% [8]. The results presented here suggest that utilising the Cherenkov light produced during treatment could provide a more accurate and cost-effective alternative. In addition to future numerical and experimental studies along the lines presented above, it would be of interest to investigate the use of Cherenkov light for 3D dosimetry. This could potentially be achieved by reconstructing the 3D distribution of Cherenkov light within the tissue using surface light measurements [10], and relating this distribution to the 3D distribution of the deposited dose using simulation and experimental calibration results such as those presented in Fig. 3– 5. This approach would also lower the error in dose estimation, in particular the error associated with the radioisotope update variability.

This study also shows that light in the NIR spectral region that can be measured at the surface originates in the treatment volume. This could be relevant for functional imaging, in conjunction with other imaging techniques such as SPECT. Assuming that the radionuclide concentration at the treatment site can be accurately determined, one could, in principle, determine (via Monte Carlo simulations, for instance) the intensity of the emitted light, which can then be combined with surface measurements to determine the optical properties of the tissue. Probing the tissue with Cherenkov light, rather than with external light sources, presents advantages in terms of accessing the tissue of interest [63]. This, however, requires novel reconstruction algorithms based on internal (rather than surface) light sources.

## Data Availability

Data underlying the results presented in this paper are not publicly available at this time but can be obtained from the authors upon reasonable request.
